# Fetal Gas Gangrene: A Rare and Critical Case

**DOI:** 10.7759/cureus.61833

**Published:** 2024-06-06

**Authors:** Bradley Chatterton, Bhavana Devanabanda, Arka Dutta, Jay Patel, Pardeep K Mittal

**Affiliations:** 1 Internal Medicine, Philadelphia College of Osteopathic Medicine, Philadelphia, USA; 2 Diagnostic Radiology, RWJBarnabas Health, Livingston, USA; 3 Radiology, Augusta University Medical College of Georgia, Augusta, USA

**Keywords:** escherichia coli, computed tomography (ct ), fetus, fetal gas gangrene, gas gangrene

## Abstract

Gas gangrene is a lethal necrotic infection resulting in gas production within tissue. It is typically associated with trauma and is especially lethal during pregnancy, resulting in severe maternal infection and fetal death. We report the case of a 31-year-old G3P2 female who presented to the emergency department with abdominal bloating, vaginal cramping, and brown vaginal discharge. Physical examination showed that the patient was hypertensive, tachycardic, and tachypneic, and laboratory examination showed a downtrending beta-human chorionic gonadotropin and leukocytosis, with elevated inflammatory markers. Ultrasound showed copious gas located within the lower abdomen and the fetus was not visualized. Computed tomography (CT) of the abdomen and pelvis showed a gravid uterus with a single fetus and extensive air locules in the fetus, amniotic cavity, and placenta. The findings were consistent with gas gangrene of a mature fetus in the third trimester. Fetal gas gangrene is a potentially lethal condition during pregnancy, and early diagnosis is imperative in management. CT was utilized in this case to outline the increased gas production within the amniotic cavity and fetal organs and proved crucial in determining the next steps of management.

## Introduction

Gas gangrene is a highly lethal, rapidly progressing necrotic infection resulting in the formation of gas within tissue [1]. It is commonly associated with high-velocity injuries related to trauma, though it can rarely arise without a history of trauma [2]. In the United States, the incidence of gas gangrene is approximately 1 in 1,000 cases per year. This figure is believed to be higher in countries with decreased access to healthcare, although the exact figures are unknown [2]. Gas gangrene develops when an organism enters through a point of injury, allowing for the proliferation of gas-forming bacteria, and decreased tissue oxygenation resulting in tissue necrosis [1-3]. If not diagnosed and treated promptly, it can spread rapidly along tissue planes resulting in sepsis [1,2]. Gas gangrene during pregnancy is an especially serious complication. Approximately two-thirds of all cases are apparent following pregnancy terminations, while the remainder are observed during term deliveries or the immediate postpartum period [3,4]. Here, we present the case of a patient with fetal gas gangrene resulting in the delivery of a non-viable fetus.

## Case presentation

A 31-year-old Hispanic female G3P2 with a past medical history of hypertension (before and during pregnancy), morbid obesity with a body mass index of 71 kg/m^2^, and preeclampsia presented to the emergency department with complaints of abdominal bloating, vaginal cramping, and brown vaginal discharge. She denied the passage of blood clots. The patient reported irregular frequency of periods and did not know she was pregnant throughout her prior pregnancies until delivery. The patient denied prior infections. She had preeclampsia with severe features in both pregnancies and was on magnesium sulfate both times. Her menarche was at the age of eight years and she had irregular menstrual periods. She visited an outside hospital the day prior with similar complaints. She was found to have a beta-human chorionic gonadotropin (beta-hCG) of 5,179 mIU/mL. The patient could not tolerate a transvaginal ultrasound (US) due to pain and left against medical advice.

The patient presented to our hospital due to continued symptoms and a desire for further workup. She was found to be hypertensive at 176/107 mmHg, tachycardic at 120 beats/minute (60-100 beats/minute), tachypneic at 24 breaths/minute (12-20 breaths/minute), and a temperature of 99.3°F (96.8-99.5°F). Routine laboratory investigation in the emergency department is shown in Table 1.

**Table 1:** Emergency department laboratory results. beta-hCG: beta-human chorionic gonadotropin; WBC: white blood cell; CRP: C-reactive protein; ESR: erythrocyte sedimentation rate

Laboratory test	Patient’s result	Reference range
Beta-hCG	2,756 mIU/mL	<5 mIU/mL
WBC count	24.4 × 10^9^/L	4.5–11.0 × 10^9^/L
Anion gap	24.3 mEq/L	4–12 mEq/L
Blood urea nitrogen	41.2 mg/dL	5–20 mg/dL
Creatinine	3.85 mg/dL	0.6–1.1 mg/dL
CRP	44.68 mg/dL	1.0–10.0 mg/dL
ESR	114 mm/hour	0–19 mm/hour

**Figure 1:**
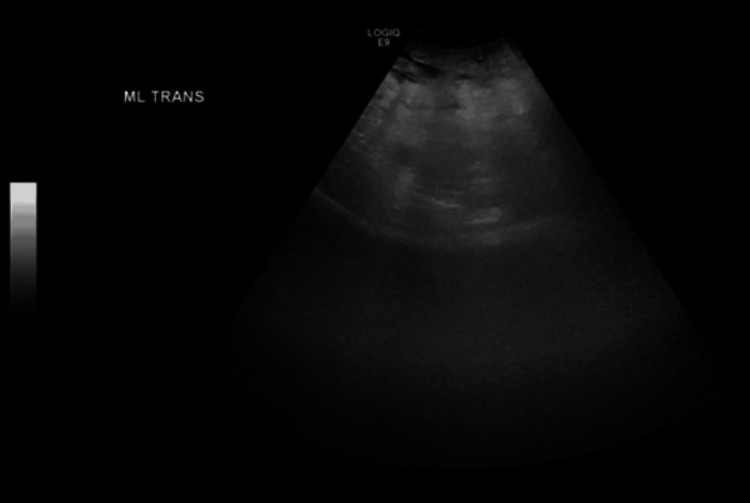
Transverse transabdominal ultrasound image demonstrates a large amount of gas in the lower abdomen limiting the visualization of the pelvic structures due to acoustic shadowing causing a ring-down artifact.

**Figure 2:**
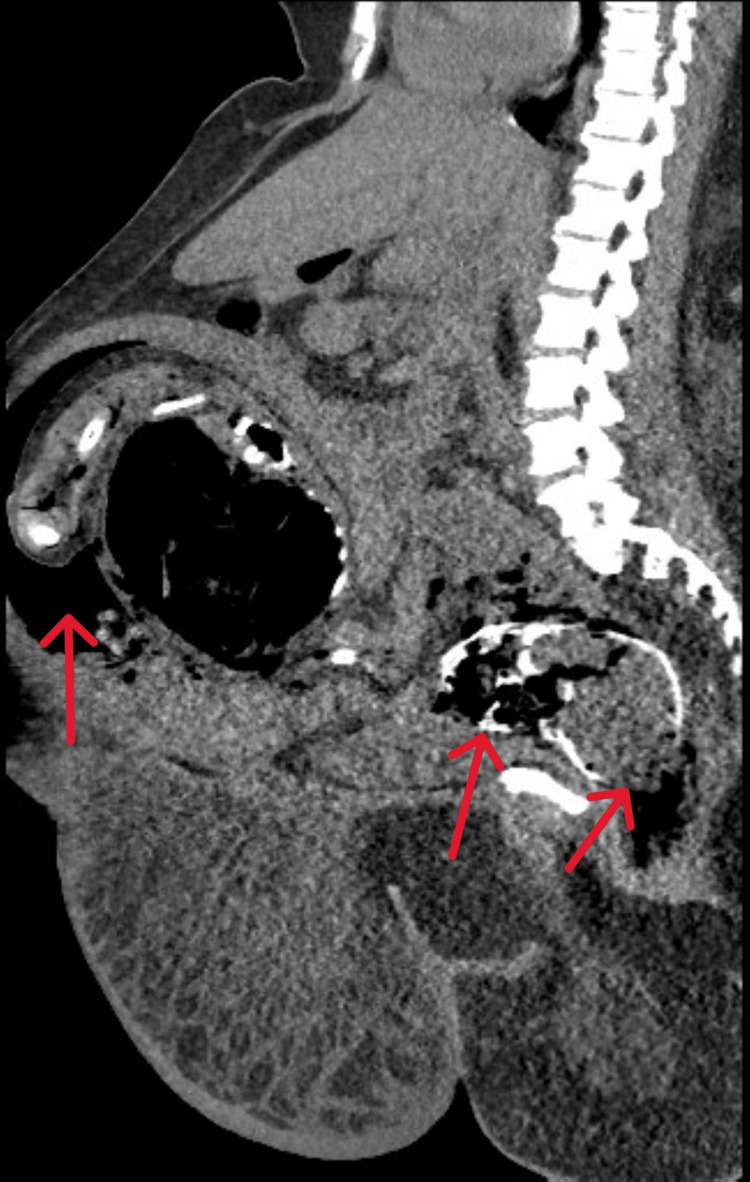
CT of the abdomen and pelvis without contrast sagittal view. Gravid uterus with the fetus measuring up to 36 cm from the calvarium to the lower pelvis. There is air in the abdominal cavity, the umbilical cord, and placental vessels (left arrow). There are extensive gas bubbles in the subdural space of the skull, thoracic cavity, abdominal cavity, and anterior soft tissue of the head and neck (middle arrow). There is an acute fracture of the fetal calvarium and there are adjacent large locules of air in the maternal vaginal canal (right arrow). The fetal bowel is dilated.

The patient presented with a clinical picture of sepsis in the setting of abnormal downtrending beta-hCG levels with brown vaginal discharge, and septic abortion was suspected. The patient was started on broad-spectrum intravenous antibiotic piperacillin-tazobactam (3.375 g IV q6) and vancomycin (1,000 mg IV q12). A transabdominal US showed copious amounts of gas (Figure 1). The patient was unable to tolerate a transvaginal US secondary to pain and was not compatible with magnetic resonance imaging (MRI) due to body habitus. Computed tomography (CT) of the abdomen and pelvis showed a gravid uterus with a single fetus in the cephalic position with extensive air locules in the fetus, amniotic cavity, and placenta. Fractures were seen in the fetal calvarium and long bones (Figure 2). The patient denied any recent trauma, and there were no signs of cervical trauma on the examination. The findings were consistent with gas gangrene of a mature fetus in the third trimester of approximately 35 weeks gestation by the crown-rump length. She underwent a uterine evacuation via vaginal delivery and became hypotensive following delivery. A central line was placed and the patient was immediately started on vasopressor therapy, which was weaned by the third day following delivery, as well as aggressive hydration therapy. A severely hydropic fetus was delivered with the placenta. Following the delivery of the non-viable fetus, a foul smell and brown vaginal discharge were noted suggestive of an anaerobic infection. On gross examination, there was a global distortion of the anatomy and sloughing of the skin. There was also a frothy, foul-smelling discharge. Peripheral blood culture was positive for methicillin-resistant *Staphylococcus epidermidis* and anaerobic placental cultures were positive for *Escherichia coli*. The patient was discharged during her 10-day antibiotic regimen with improving symptoms. Data was not available regarding outpatient follow-up.

## Discussion

Fetal gas gangrene is a rare and lethal complication in pregnancy. There are very few reported cases in developed countries. Most patients undergo antenatal testing for infectious diseases to prevent intra-amniotic infections. Most cases of fetal gas gangrene are seen in patients with poor prenatal health checks.

There are two primary ways bacteria can reach the fetus. Traumatic entry via premature rupture of membranes, which introduces organisms directly into the uterus, or spontaneously through hematogenous spread from the mother. Anaerobic bacteria such as *Clostridium perfringens*, *Clostridium septicum*, *Prevotella brevia*, *Bacteroides capillosus*, and *Anaerococcus prevotii* have been previously reported [4,5]. The placental cultures in our patient grew *Escherichia coli*. Once the bacteria seeds into the dead fetal tissue, gas is produced through anaerobic respiration [1,2]. The bacteria can spread to the surrounding endometrium and cause endometritis. It can progress to infect the myometrium and enter the peritoneal cavity through the fallopian tube. Maternal hematogenous spread can result in a cytokine surge that can trigger multiorgan shock and disseminated intravascular coagulation [6].

Early clinical manifestations are fever, cramping lower pelvic and back pain, and foul-smelling brown or red vaginal discharge. Physical examination may present with abdominal wall crepitus [7]. Late clinical manifestations can present as systemic inflammatory response, hemodynamic instability, multiorgan failure, and disseminated intravascular coagulation [5,7]. Therefore, early diagnosis and intervention are vital. Fetal gas gangrene can be diagnosed with US which evaluates anatomic structures and fetal heart rate. Sometimes large amounts of gas can cause shadowing that limits the evaluation of the fetus. MRI can also be used as there is no risk of radiation exposure to the mother and fetus [1]. CT is used as a last resort for diagnosis due to radiation exposure.

There have been few previously reported cases of prenatal CT scan diagnosis of gas gangrene, including cases by Abe et al. and Venkatesha Gupta et al. [4,7]. CT scan is a reliable method to identify emphysematous changes caused by gas gangrene in the amniotic cavity afflicting the fetus [7]. The prognosis of fetal gas gangrene depends on early intervention. Fetal gas gangrene is managed by immediate vaginal delivery. Labor is initiated with uterotonic agents such as intravenous oxytocin [1]. Treatment of gas gangrene requires antibiotic therapy aimed to quell invading organisms, supportive therapy to address organs damaged by toxins produced by bacteria, and surgical debridement if severe [3,8]. Empirically, broad-spectrum antibiotics are initiated and then replaced by narrow-spectrum antibiotics based on the culture sensitivities [1,8]. In our case, transabdominal imaging yielded poor-quality images that mainly demonstrated gas. The patient was not able to tolerate a transvaginal US due to immense pain. MRI was not an option because of the patient’s body habitus. The risks and benefits of a CT scan were explained to the patient, and she agreed to proceed. CT scan (Figure 2) showed subcutaneous, intra-abdominal, intrathoracic, and intracranial emphysema in the fetus. The skull was deformed, and intracranial contents had spilled into the amniotic cavity. The bones were deformed or fractured, a feature not previously reported in the literature, and is likely a result of the aggressive nature of the infection. The patient delivered the non-viable fetus and was administered piperacillin-tazobactam along with vancomycin antibiotic treatment and supportive measures.

## Conclusions

Gas gangrene is a rare and lethal complication in pregnancy. Early diagnosis is critical in the management and can be done with US, MRI, or CT. Upon diagnosis, the patient should undergo immediate delivery and empiric antibiotic treatment, which can be further narrowed with culture sensitivities. However, further studies are needed to establish the best diagnostic approach and subsequent management of this condition.
